# Society Meetings

**Published:** 1899-09

**Authors:** 


### SOCIETY MEETINGS.

The American Electro-therapeutic Association will hold its next annual meeting September 19, 20 and 21, 1899, at Washington, D. C., under the presidency of Dr. F. B. Bishop. Many electrical manufacturers will be represented in the Exhibit Hall, and information will be freely given. Papers have thus far been promised from Drs. R. G. Nunn, A. D. Rockwell, Margaret A. Cleaves, F. J. Leviseur, Walter White, Robert Reyburn, G. A. Corson, C. O. Files, J. H. Kellogg, John A. Licthy, W. W. Scheppegrell, L. Howe, E. Wende, F. B. Bishop, Robert Newman, W. J. Herdman, G. B. Massey and Professors Bergonie, of Bordeaux; Apostoli and Dolbear, of Paris. The chairman of the press committee is Dr. Llewellyn Eliot.

---

The annual meeting of the Lehigh Valley Railroad Surgeons was held at the International Hotel, at Niagara Falls, July 21, 1899. The following papers were read and discussed : President's address, Consideration of railroad injuries, with special reference to suits for damages based on so-called railroad spine, Dr. W. G. Weaver, Wilkes-Barre, Pa.; Some practical points in the management of injuries at the elbow joint, Dr. H. T. Dana, Cortland, N. Y.; Treatment of burns, Dr. G. W. Farquhar, Pottsville, Pa.

The following officers were elected for the ensuing year : President, C. H. Ott, Sayre, Pa.; first vice-president, L. E. Hollister, Jersey City; second vice-president, O. E. McCarty, Niagara Falls; third vice-president, P. Hermany, Mahanoy City, Pa.; secretary and treasurer, J. G. Zem, Lehighton, Pa.; executive committee, W. L. Estes, surgeon-in-chief, South Bethlehem, Pa., ex-officio; R. H. Wilbur, general superintendent, South Bethlehem, Pa., ex-officio; J. G. Zem, secretary and treasurer, Lehighton, Pa., ex-officio; E. B. Dana, Metuchen, N. J.; W. T. Williams, Mount Carmel Pa.; W. R. Longshore, Hazleton, Pa.; A. Stout, Bethlehem, Pa. Delegates to International Association, W. G. Weaver, Wilkes-Barre, Pa.; O. E. McCarty, Niagara Falls.

After the meeting the members with their wives and families visited points of interest about the Falls, after which a ride on the trolley down the Canadian side and the return through the Gorge was taken. Thirty-one members were present and the meeting proved one of the most successful ever held by the association. Dr. G. R. Trowbridge, of Buffalo, and Dr. O. E. McCarty, of Niagara Falls, were the committee of arrangements. The meeting next year will be held in New York City.

---

OFFICERS of the Buffalo Academy of Medicine—Session 1899-1900: President, Matthew D. Mann; secretary, Thomas F. Dwyer; treasurer, Eugene A. Smith; trustees, Stephen Y. Howell, three years; Benjamin G. Long, two years; Marcel Hartwig, one year. The vice-presidents by virtue of being chairmen of the various sections are: Eli H. Long, chairman section on medicine; J. Henry Dowd, chairman section on surgery; Henry D. Ingraham, chairman section on obstetrics and gynecology; Arthur G. Bennett, chairman section on pathology. The council is, therefore, composed of the aforementioned officers, the president of the Academy of the previous year, Roswell Park, and the secretaries of the sections, who are: J. C. Clemesha, secretary section on medicine; Marshall Clinton, secretary section on surgery; Albert J. Colton, secretary section on obstetrics and gynecology; Frederick H. Millener, secretary section on pathology.

PROGRAM, 1899–1900.

September 5th, Surgery—American aborigines, from a medical and surgical standpoint, A. L. Benedict.

September 12th, Medicine—Quarterly meeting of the Academy—The medical aspect of crime, Daniel R. Brower, professor of mental disease and therapeutics, Rush Medical College, Chicago.

September 19th, Pathology—subject to be announced, George M. Gould, Philadelphia, Pa. Exhibition of microscopic slides illustrating glaucoma, R. H. Satterlee. Subject to be announced, Edmund E. Blaauw.

September 26th, Obstetrics—The relation of gastric disturbances to gynecologic diseases, A. L. Benedict. Anesthesia, Francis W. McGuire. Ectopic pregnancy, L. E. McC. Pomeroy.

October 3d, Surgery—Subphrenic abscess, Prescott LeBreton. Early diagnosis and treatment of tumors of the breast, L. E. McC. Pomeroy.

October 10th, Medicine—Malaria, Irving P. Lyon; discussion by Charles G. Stockton, Marshall Clinton and John D. Howland.

October 17th, Pathology—Landry's paralysis, Dewitt H. Sherman.

October 24th, Obstetrics—Subject to be announced, R. L. Banta. Pus in the pelvic cavity, Herman E. Hayd.

November 7th, Surgery—Subject and essayist to be announced. Subject to be announced, Herman Mynter.

November 14th, Medicine—Protracted pneumonia and slow resolution, John H. Pryor. Hygiene of the asthmatic, George N. Jack, Depew, N. Y.

November 21st, Pathology—The relation of dentition to disease, C. S. Butler, D. D. S.

November 28th, Obstetrics—Subjects to be announced, P. W. VanPeyma; Earl P. Lothrop.

December 5th, Surgery—Quarterly meeting of the Academy—Subject and essayist to be announced. Vesicated suprarenal capsule in acute coryza, Frederick H. Millener.

December 12th, Medicine—The relation of indicanuria to secretion of hydrochloric acid, Allen A. Jones. Addison's disease, with a report of a case, Helene Kuhlmann.

December 19th, Pathology—The ear, George F. Cott. Subject to be announced, Max Keiser.

December 26th, Obstetrics—Subject to be announced, Charles E. Congdon. The difficulties a young doctor encounters in obstetric practice, Nelson W. Wilson.

January 2d, Surgery—Subject to be announced, Chauncey P. Smith. Hypertrophy of the prostate, Charles F. Durand.

January 9th, Medicine—Typhlitis and scolecitis, A. L. Benedict. The treatment of acute appendicitis, DeLancey Rochester.

January 16th, Pathology—Lawson Tait, Stephen Y. Howell. Subject to be announced, Herman E. Hayd.

January 23d, Obstetrics—Differential diagnosis of disease of the appendix and Fallopian tubes, C. C. Frederick. Subject to be announced, Walter D. Green. Eruptive diseases of the female genitals, J. Henry Dowd.

February 6th, Surgery—Subject and essayist to be announced.

February 13th, Medicine—Dress reform, Matthew D. Mann and Mrs. Mankell.

February 20th, Pathology—Extra genital syphilis, Grover W. Wende. New method of analysis of stomach contents, A. L. Benedict.

February 27th, Obstetrics—Title to be announced, Edward J. Ill, Newark, N. J.

March 6th, Surgery—Subject to be announced, Roswell Park. Goitre, Edward J. Meyer.

March 13th, Medicine—Senile bronchitis, Reynold W. Wilcox, professor of medicine and therapeutics, New York Post-graduate Medical School.

March 20th, Pathology—Quarterly meeting of the Academy—Subject to be announced, Frederick Peterson, New York. Subject to be announced, Thomas B. Carpenter.

March 27th, Obstetrics--The early days of ovariotomy; reminiscences of McDowell, Peasley, etc., Matthew D. Mann. Subject to be announced, Helene Kuhlmann.

April 3d, Surgery—Subject and essayist to be announced.

April 10th, Medicine—The treatment of scarlet fever, Henry R. Hopkins. Normal saline solution in gastro-enteric diseases of children, Irving M. Snow.

April 17th, Pathology—Our present knowledge of the inflammatory process, H. R. Gaylord. Subject to be announced, Charles S. Jewett.

April 24th, Obstetrics—Asepsis and antisepsis in obstetric practice, William Warren Potter. Subject to be announced, L. Bradley Dorr. An unusual case of asphyxia, Thomas F. Dwyer.

May 1st, Surgery—Subject to be announced, Edward M. Dooley. Adjustment of glasses for young children, Arthur G. Bennett.

May 8th, Medicine—Gonorrheal arthritis, J. Henry Dowd.

May 15th, Pathology—Subjects to be announced, Herbert D. Pease, State Pathological Laboratory; Charles E. Congdon.

May 22d, Obstetrics—Asepsis and anti-asepsis in obstetrics, Devillo W. Harrington. Obstetrical reminiscences, James S. Smith; discussion of both papers by C. C. Wyckoff, Samuel G. Dorr, S. S. Green and others.

June 5th, Surgery—Subject to be announced, Charles E. Congdon.

June 12th—Annual meeting of the Academy.

June 19th, Pathology—Subject to be announced, Herbert U. Williams.

June 26th, Obstetrics—Quarterly meeting of the Academy—Subjects to be announced, M. A. Crockett; Lillian C. Randall; Maud J. Frye.

---

The Medical Society of the County of Chautauqua held its annual meeting at the hotel Atheneum, Chautauqua, N. Y., July 11, 1899, Dr. Morris N. Bemus presiding. The following officers were elected for the next year: President, V. M. Griswold, Fredonia; vice-president, E. A. Scofield, Bemus Point; secretary, C. A. Ellis, Sherman.

The semiannual meeting will be held at Jamestown. The program was carried out with but one exception and the attendance was the largest that has ever been known. Dr. Morris illustrated his paper with a manikin and Dr. Seaver had present Mr. Sheldon, of Erie, Pa., who had worn a spinal brace over forty years of his own manufacture, and one that seemed to be ideal. The evening paper was illustrated by stereopticon lights and was given in the spacious parlors of the hotel.

Dr. Jane Greeley, of Jamestown; Drs. C. W. Southworth and V. D. Bozovsky, of Dunkirk and Dr. Francis Hunt, Dewittville, were elected to membership.

---

More than 100 international congresses of various kinds are to be held in Paris next year during the course of the exposition. A certain number of these of more special medical interest have already been announced in the columns of the *Medical News*. Just about the time of the International Medical Congress, that of from August 2d to 9th, the following congresses on subjects of more or less importance to medical men are to be held: An International Congress of Applied Chemistry from July 23d to the 31st; International Congress for the Amelioration of the Condition of the Blind, August 5th; International Congress of Public Charities and Philantrophy, July 30th to August 5th; International Congress of Deaf Mutes during the first week in August; International Congress of the Medical Press sometime during Medical Congress week; International Dental Congress, August 8th to 14th; International Congress of Professional

Relations and Deontology (the science of duties) from July 23d to 28th; International Congress of Psychology, August 22th to 25th; International Congress of Hygiene, August 10th to 17th.—*Medical News.*

---

THE last regular meeting of the Niagara Falls Academy of Medicine was held Monday, August 7th, at 8.45 p. m., at the office of Doctor F. R. McBrien, 113 Falls street. A paper was read by Dr. Scott on the Obstetrical forceps.

---

THE Association of Military Surgeons of the United States will hold its annual meeting at Kansas City, Mo., September 27, 28 and 29, 1899, under the presidency of Major J. D. Griffith, M. D., U. S. V., of Kansas City. Major James E. Pilcher, M. D., U. S. Army, of Carlisle, Pa., is the secretary.

